# Phase-Change Memory Properties of Electrodeposited Ge-Sb-Te Thin Film

**DOI:** 10.1186/s11671-015-1136-4

**Published:** 2015-11-02

**Authors:** Ruomeng Huang, Gabriela P. Kissling, Andrew Jolleys, Philip N. Bartlett, Andrew L. Hector, William Levason, Gillian Reid, C. H. ‘Kees’ De Groot

**Affiliations:** School of Electronics and Computer Science, University of Southampton, Southampton, SO17 1BJ UK; School of Chemistry, University of Southampton, Southampton, SO17 1BJ UK

**Keywords:** Phase-change memory, Electrodeposition, Ge-Sb-Te

## Abstract

We report the properties of a series of electrodeposited Ge-Sb-Te alloys with various compositions. It is shown that the Sb/Ge ratio can be varied in a controlled way by changing the electrodeposition potential. This method opens up the prospect of depositing Ge-Sb-Te super-lattice structures by electrodeposition. Material and electrical characteristics of various compositions have been investigated in detail, showing up to three orders of magnitude resistance ratio between the amorphous and crystalline states and endurance up to 1000 cycles.

## Background

The development of denser, faster and less energy-consuming non-volatile memory (NVM) is critical to innovations in modern information technology [1]. Among all the competitors for the next generation of NVM, chalcogenide-based phase-change memory (PCM) is one of the most promising candidates for its advantages of high speed, high scalability, high endurance, low power consumption and good compatibility with the complementary metal-oxide semiconductor (CMOS) process [2–4]. Data storage in PCM is achieved by rapidly switching the phase-change material between its amorphous (high-resistance) state and crystalline (low-resistance) state with Joule heating. Materials within the Ge-Sb-Te (GST) ternary phase diagram are generally regarded as suitable phase-change materials, with Ge_2_Sb_2_Te_5_ being the most popular material in this application. The recent development of GeTe/Sb_2_Te_3_ super-lattice structure-based interfacial phase-change memory data storage devices has shown even better performance with reduced switching energies, improved write-erase cycle lifetimes and faster switching speeds [5, 6]. The conventional method for the growth of crystalline super-lattice structures using molecular beam epitaxy (MBE) requires a nearly perfect match between the lattice constants of the two materials to allow epitaxial growth, which limits the selection of the super-lattice components. Amorphous GeSbTe super-lattices have been grown previously using ion beam deposition [7]. Unlike MBE-grown crystalline super-lattices, the amorphous super-lattices have no epitaxial or lattice matching [8], but might still provide many of the advantages in particular related to reducing power in switching of phase-change memory.

Common methods for the deposition of phase-change materials use physical vapour deposition (PVD), chemical vapour deposition (CVD) or atomic layer deposition (ALD) [9–12]. Comparing with those conventional techniques, electrodeposition offers several potentially significant advantages for the growth of semiconductor alloys [13–16]. It is a fast and lower cost alternative to the vapour deposition techniques as it does not require ultra-high vacuum (UHV) equipment or high temperatures, and it can fill high-aspect-ratio cells. Several attempts have been made to grow phase-change materials by electrodeposition. Notable results were reported for the electrodeposition of binary BiTe and SbTe films [17, 18]. However, incorporating the electrochemically challenging germanium for the electrodeposition of ternary GeSbTe materials has proved to be extremely difficult in conventional aqueous solution. We recently reported a new method for the electrodeposition of amorphous ternary GeSbTe materials from a single, highly tuneable, non-aqueous electrolyte [19]. This approach enables excellent control over the composition across the ternary phase diagram and also allows the selective deposition into nanostructures [20].

In this paper, we report the properties of a series of electrodeposited GeSbTe compounds with various compositions. These compositions can be achieved by varying either the precursor concentrations in the electrolytes or the deposition potentials in a single electrolyte. The latter method opens up the prospect of depositing super-lattice structures by electrodeposition, as has been previously shown for metallic compounds [21, 22]. The electrodeposited phase-change memory cells which have been fabricated are characterised in terms of the on/off ratio of the amorphous and crystalline states, threshold voltage and cyclability.

## Methods

### Electrochemical Preparation

Three precursors, [N^n^Bu_4_][GeCl_5_], [N^n^Bu_4_][SbCl_4_] and [N^n^Bu_4_]_2_[TeCl_6_], were prepared and purified for the ternary electrodeposition. All electrochemical experiments and the electrolyte preparation were performed inside a glove box in order to exclude moisture and oxygen (<5 ppm). Electrolytes were prepared in anhydrous CH_2_Cl_2_ using 0.1 mol dm^−3^ tetrabutylammonium chloride ([N^n^Bu_4_]Cl) as the supporting electrolyte. A Pt gauze was used as the counter electrode, and for the reference electrode, a home-made Ag/AgCl reference electrode, using 0.1 mol dm^−3^ [N^n^Bu_4_]Cl in CH_2_Cl_2_, was used. All experiments were performed in a standard one-compartment electrochemical cell within a wire mesh Faraday cage.

### Thin-Film Characterisation

The deposited GST films were investigated using scanning electron microscopy (SEM) and energy-dispersive X-ray spectroscopy (EDX). A Zeiss EVO LS 25 microscope equipped with an Oxford INCA x-act X-ray detector was used for the SEM and EDX analyses. For EDX quantification measurements, a sputtered thin film from a Ge_2_Sb_2_Te_5_ target was used for calibration. High-resolution SEM measurements were carried out with a field emission SEM (Jeol JSM 7500F). Films were imaged in high-vacuum mode. Annealing was performed using a rapid thermal annealer (Jipelec JetFirst) in a N_2_ atmosphere.

### Memory Cell Fabrication and Characterisation

Electrodeposition was performed on planar TiN substrates in order to provide a technologically relevant substrate. A 200-nm TiN film was deposited onto a SiO_2_/Si substrate by reactive sputtering at room temperature. After the electrodeposition of GeSbTe films, another TiN film was sputtered on top of the GeSbTe films and subsequently patterned by photolithography and plasma etching to form the top electrode of the memory cell.

All electrical measurements were performed with a Keithley 4200 semiconductor characterisation system. During the measurements, the programming voltage bias was applied to the top electrode, while keeping the bottom electrode grounded. The probe/point contacts to the top and bottom electrodes of the devices were realised through a pair of Wentworth probe needles, using a Wentworth Laboratories AVT 702 semi-automatic prober. For electrical pulsing, two ns-pulsing measuring units (PMUs) were integrated within the same characterisation setup. The resistance of the devices after applying set or reset voltage pulses was measured at 0.1 V (DC).

## Results and Discussion

### Potential-Dependent Electrodeposition

**Fig. 1:**
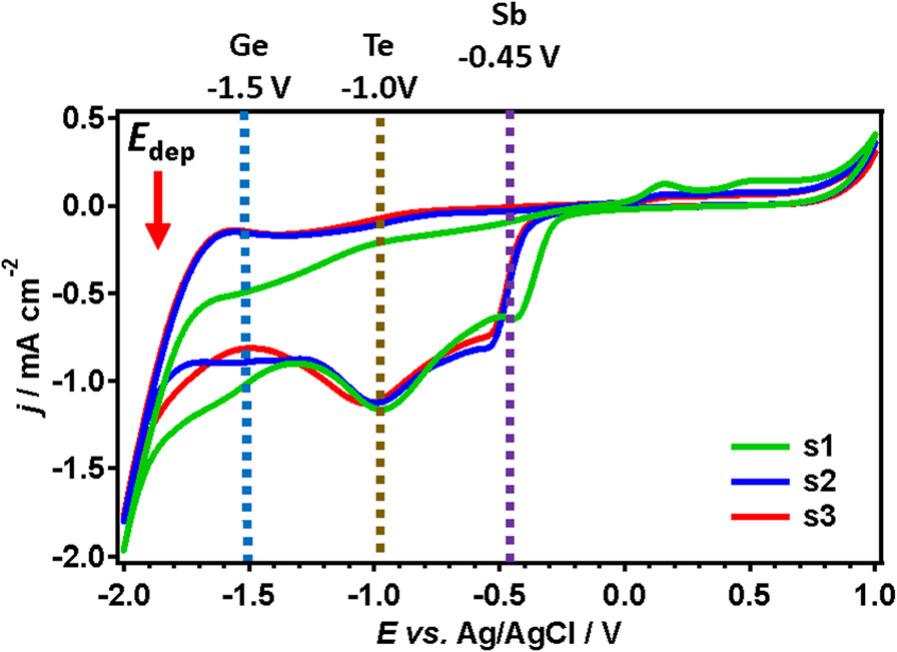
Cyclic voltammograms of the combined electrolyte recorded on glassy carbon. The electrolyte contained 1:1:2 mM dm^-3^ Ge:Sb:Te; the three observed waves are associated with the three precursors

**Fig. 2:**
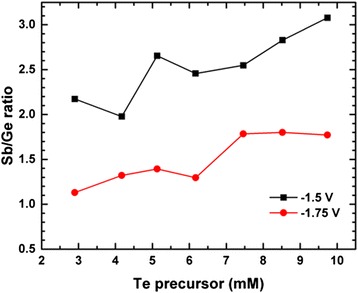
Sb/Ge compositional ratio in the resultant films from two different deposition potentials as a function of Te precursors concentration in different electrolytes

Figure 1 depicts the typical cyclic voltammograms with three consecutive scans representative for the optimised electrolyte required for Ge_2_Sb_2_Te_5_. The two peaks at approximately −0.45 and −1.0 V and the shoulder at −1.50 V correspond with the reduction peaks for the individual Sb, Te and Ge reagents, respectively. The different threshold voltage for deposition of the different materials opens up the opportunity to vary the composition of the compound by varying the deposition potential. Seven electrolytes with the same Ge and Sb concentration (10:10 mM) and varying Te concentration (3–10 mM) were deposited at two different voltages (−1.5 and −1.75 V). The compositions of the material vary from Ge_2.3_Sb_5.0_Te_2.7_ (−1.5 V) to Ge_4.0_Sb_4.5_Te_1.5_ (−1.75 V) for 3 mM Te and from Ge_0.9_Sb_2.6_Te_6.5_ (−1.5 V) to Ge_0.9_Sb_1.6_Te_7.5_ (−1.75 V) for 10 mM Te. The key effect of the deposition potential can be monitored by the Sb/Ge compositional ratio in the deposited film when the precursor ratio is constant, as shown in Fig. 2. It is clear that even with the same precursor concentration, the change of deposition potential has a significant effect on the Sb/Ge ratio. A more negative potential (−1.75 V) leads to more Ge deposition and hence a lower Sb/Ge ratio, while a decrease of the potential to −1.5 V suppresses the deposition of Ge and results in a higher Sb/Ge ratio. This is in agreement with the cyclic voltammetry which clearly shows that Ge has the more negative deposition potential and is hence not yet in the mass limited transport regime. These results are consistent for all seven electrolytes used in this work. In addition, an increasing trend of the Sb/Ge ratio can also be observed with increasing Te precursor concentrations in both cases. This indicates that the independent deposition approximation is not perfect and that subtle interplays between the precursors exist. The ability of controlled variation of the ratio of elements with deposition potential demonstrates the prospects of rapidly changing the film composition by changing the deposition potential during electrodeposition, to create super-lattice structures.

### Concentration-Dependent Electrodeposition

**Fig. 3:**
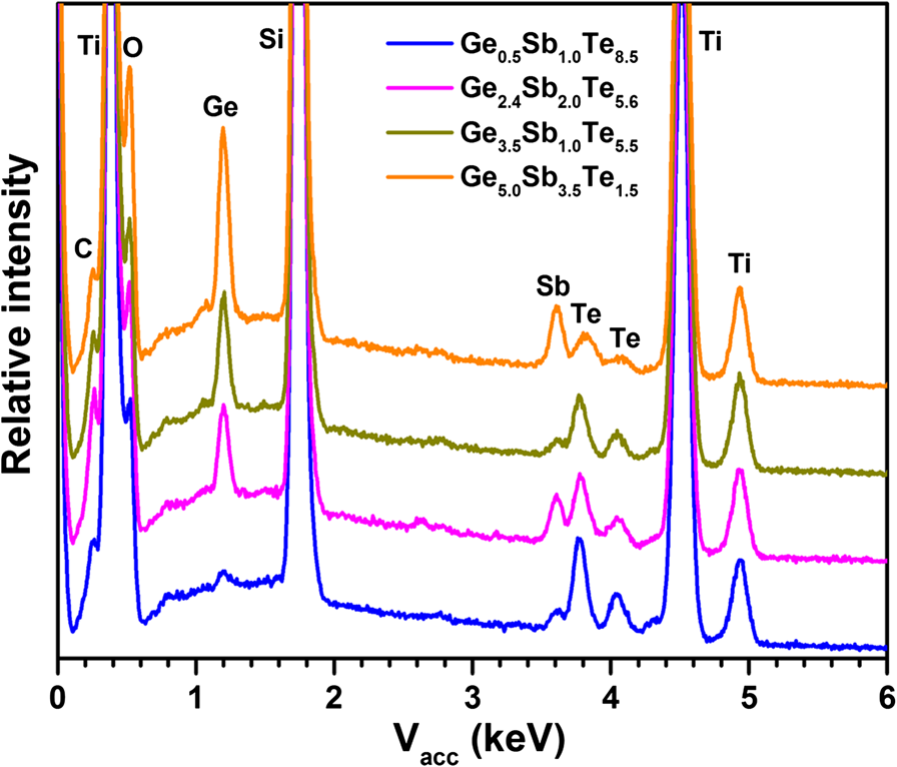
EDX spectra of the four electrodeposited GeSbTe films, normalised to the Ti peak from the underlying TiN film

In concentration-dependent electrodeposition, all depositions were performed potentiostatically at −1.75 V, more cathodic than the germanium deposition potential, to ensure significant deposition of all three elements. The film thickness was controlled by the total electrodeposition charge density of 0.4 C cm^−2^, which corresponds to a film thickness of *ca.* 200 nm. Energy-dispersive X-ray (EDX) spectra for all films are displayed in Fig. 3. The Ge, Sb and Te elements can be clearly observed in all cases, with the ratio of the main peaks of each element uniformly increasing with increasing electrolyte concentration. Other peaks arise from the TiN, Si and SiO_2_ layers, with no noticeable impurities except for some carbon content. Quantification of EDX results indicates that the film compositions are approximately (a) Ge_0.5_Sb_1.0_Te_8.5_, (b) Ge_2.4_Sb_2.0_Te_5.6_, (c) Ge_3.5_Sb_1.0_Te_5.5_ and (d) Ge_5.0_Sb_3.5_Te_1.5_, as tabulated in Table 1 against the precursor concentrations in the electrolyte. The quantification is calibrated against a sputtered Ge_2_Sb_2_Te_5_ film of the same thickness.

**Table 1:** EDX quantification results for GeSbTe films electrodeposited from different electrolytes

[N^n^Bu_4_][GeCl_5_] (vol. %)	[N^n^Bu_4_][SbCl_4_] (vol. %)	[N^n^Bu_4_]_2_[TeCl_6_] (vol. %)	Resultant compositions
8	30	62	Ge_0.5_Sb_1.0_Te_8.5_
25	25	50	Ge_2.4_Sb_2.0_Te_5.6_
46	18	36	Ge_3.5_Sb_1.0_Te_5.5_
40	40	20	Ge_5.0_Sb_3.5_Te_1.5_

**Fig. 4:**
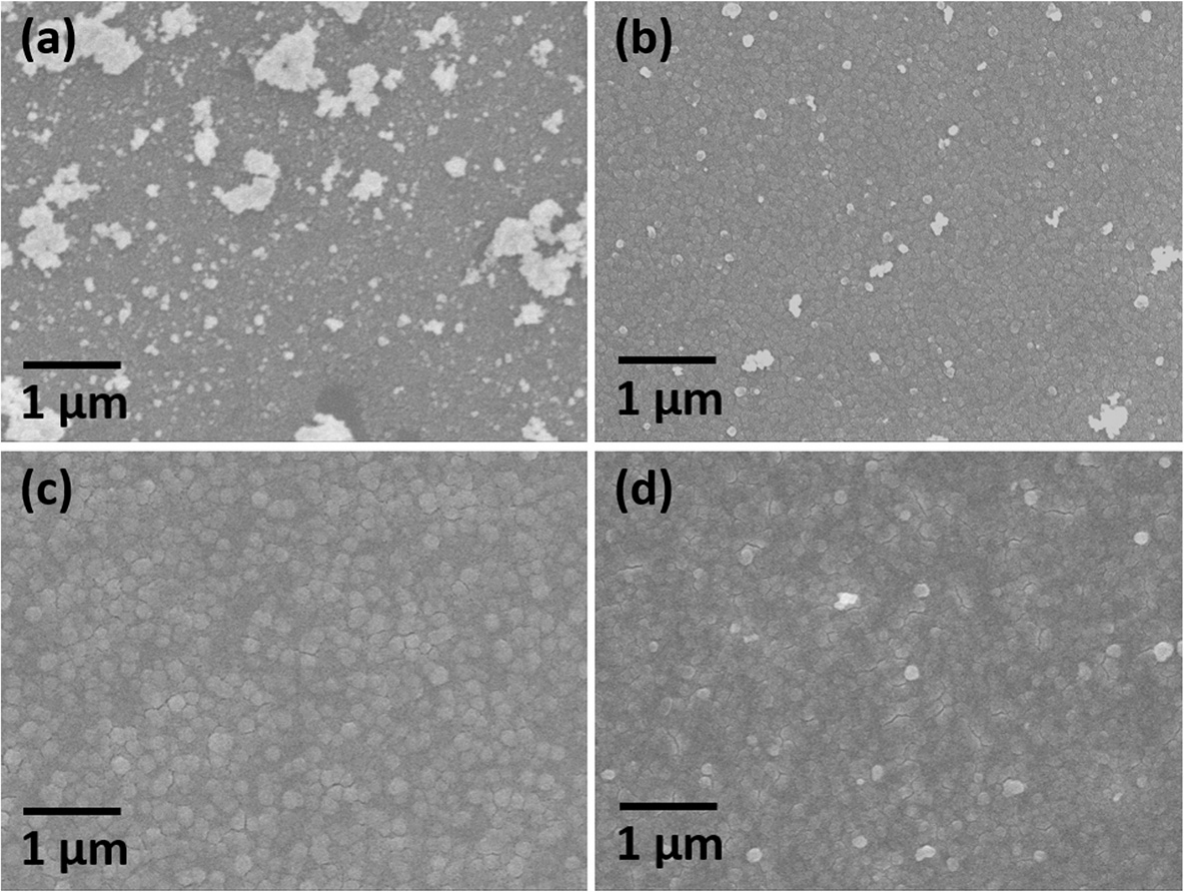
SEM images of electrodeposited phase-change memory films with composition (**a**) Ge_0.5_Sb_1.0_Te_8.5_, (**b**) Ge_2.4_Sb_2.0_Te_5.6_, (**c**) Ge_3.5_Sb_1.0_Te_5.5_ and (**d**) Ge_5.0_Sb_3.5_Te_1.5_

Top-view SEM images of the four as-deposited films are shown in Fig. 4. The Ge-poor Ge_0.5_Sb_1.0_Te_8.5_ film is characterised by small granules with noticeable irregular nucleation on the surface (Fig. 4a). The granule size increases significantly with the increase of the Ge content in the film as shown in Fig. 4b–d.

### Phase-Change Memory Cells

**Fig. 5:**
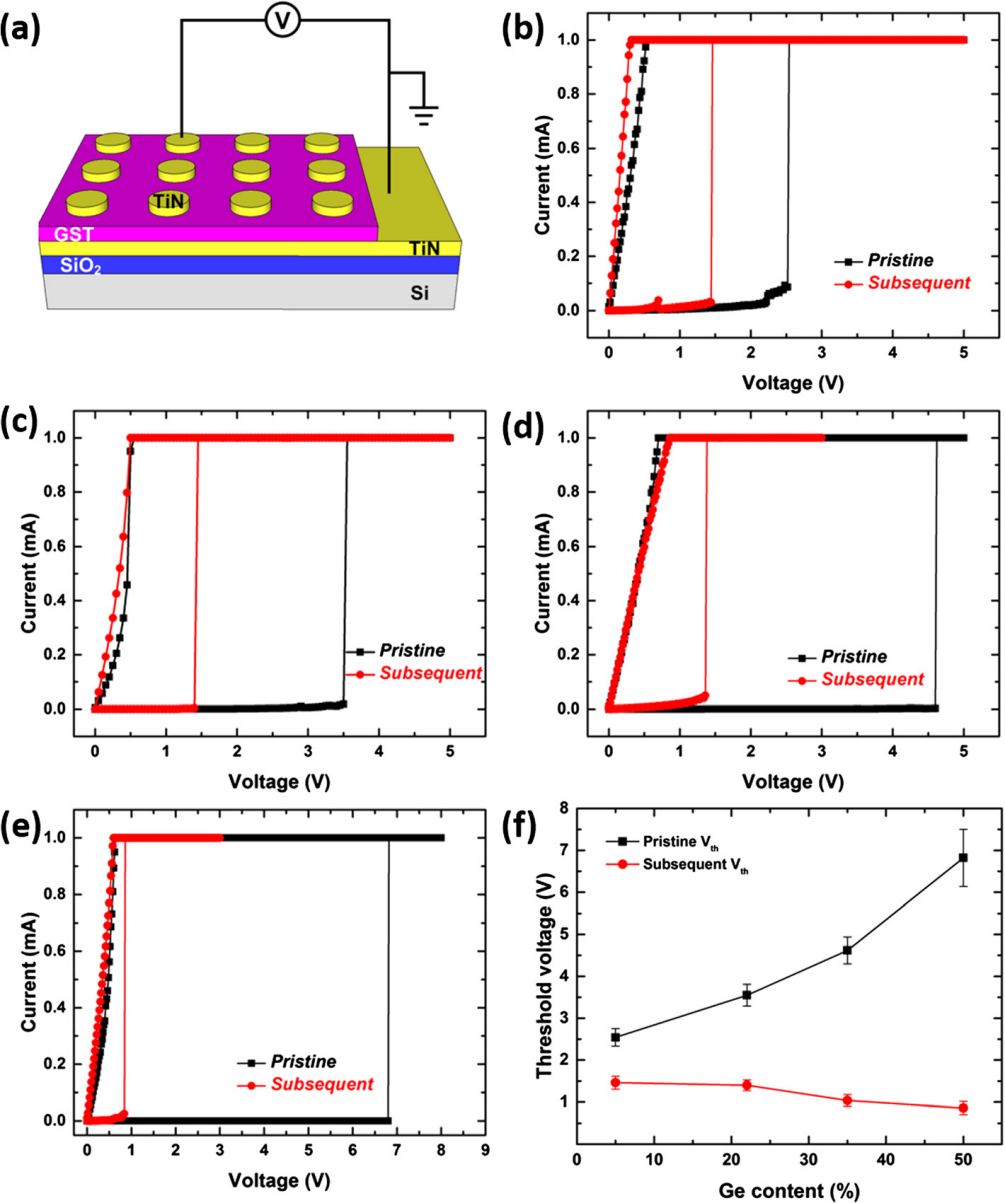
Memory switching characteristics of electrodeposited GeSbTe devices. **a** Schematic of the vertical GeSbTe memory cells and the electrical characterisation setup. DC sweep of the (**b**) Ge_0.5_Sb_1.0_Te_8.5_, (**c**) Ge_2.4_Sb_2.0_Te_5.6_, (**d**) Ge_3.5_Sb_1.0_Te_5.5_ and (**e**) Ge_5.0_Sb_3.5_Te_1.5_ memory cells. **f** Threshold voltage (*V*_th_) as a function of the Ge content in the GeSbTe films

The electrical memory switching properties of the electrodeposited GST thin films were investigated by fabricating vertical memory devices with TiN contacts on top of the GeSbTe films (Fig. 5a). The same device size (50 μm) and film thickness (200 nm) were used to allow direct comparisons between different thin-film devices. The *I*-*V* characteristics of all four as-fabricated memory devices are first studied by DC sweep as shown in Fig. 5b–e. All pristine devices display high initial resistances, indicating that the as-deposited films are in the amorphous state. For the Ge_0.5_Sb_1.0_Te_8.5_ thin-film device (Fig. 5b), this high-resistance state remains until the threshold value is reached (*ca.* 2.5 V), where the current suddenly increases to the compliance value (1 mA). The back sweep then shows linear *I*-*V* behaviour, which is consistent with the material having switched from the amorphous state to crystalline state. Similar switching features can be observed for the other three compositions. It is observed that a higher Ge content in the GeSbTe material leads to a higher threshold voltage in the memory cell, as shown in Fig. 5f. Similar behaviour was reported in [23]. Although the threshold switching mechanism for phase-change materials is still under investigation, it is generally accepted that the threshold switching field (*E*_t_) depends on the intrinsic material properties, including the optical band-gap, where the higher the optical band-gap corresponds to the higher threshold switching field [24, 25]. The Ge-poor GeSbTe materials have lower optical band-gaps [26] and hence have lower threshold voltages for a given film thickness. After the initial threshold switching, all memory cells were reset to the high-resistance state by electrical pulsing. The subsequent DC sweeps display much lower threshold voltages. This is explained by the formation of a smaller programming region within the memory cell [27]; the melt quenching in the reset process does not re-amorphize the entire film, leading to a decrease of the effective device length. However, a direct comparison of the subsequent *V*_th_ is not possible as the re-amorphized areas are different for each composition.

**Fig. 6:**
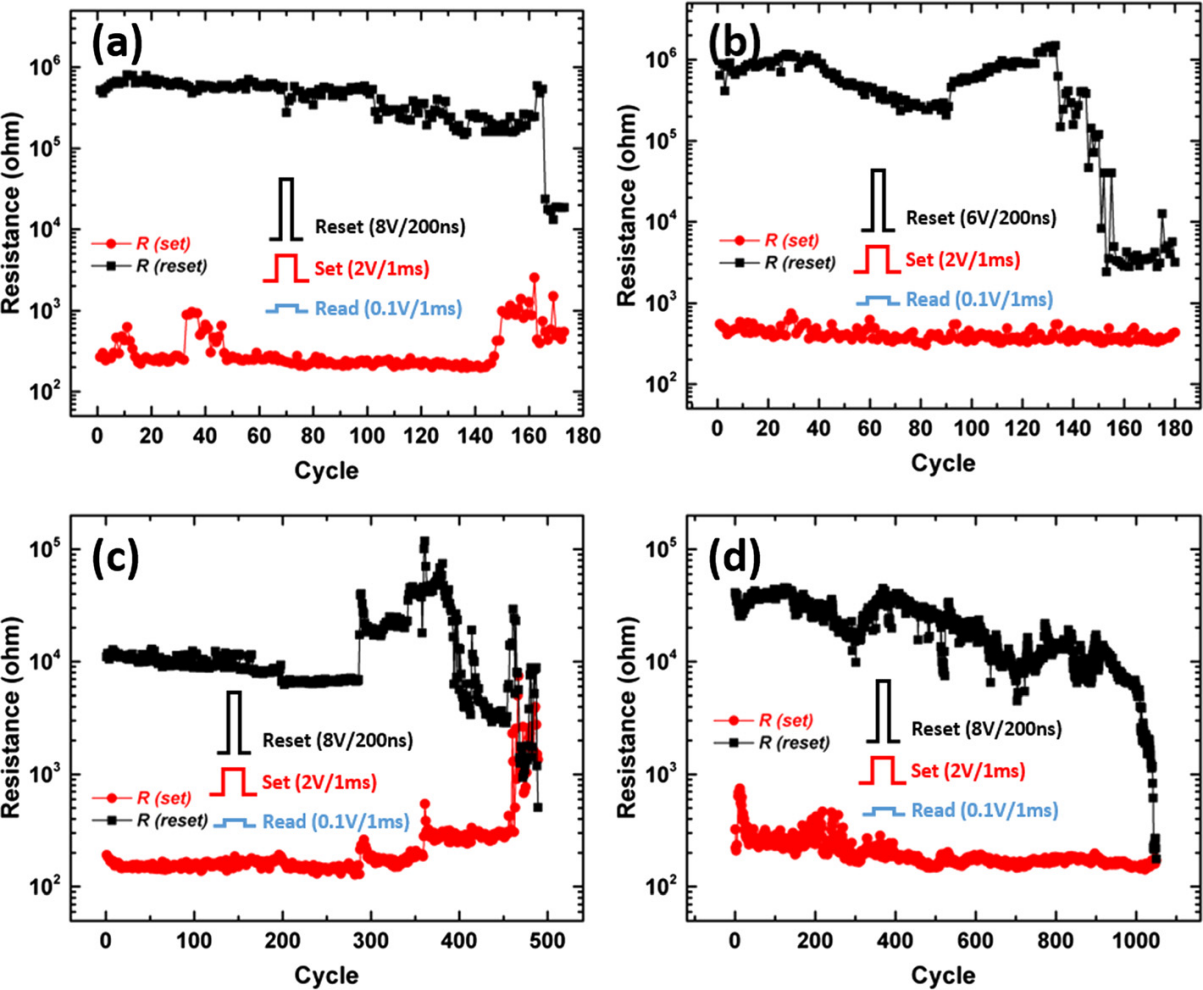
Representative endurance performance of the (**a**) Ge_0.5_Sb_1.0_Te_8.5_, (**b**) Ge_2.4_Sb_2.0_Te_5.6_, (**c**) Ge_3.5_Sb_1.0_Te_5.5_ and (**d**) Ge_5.0_Sb_3.5_Te_1.5_ vertical memory cells

The representative endurance performance of the four memory cells based on electrodeposited GeSbTe films were characterised by electrical pulsing as shown in Fig. 6. The Ge_0.5_Sb_1.0_Te_8.5_ thin-film device displays stable endurances over 100 cycles. A dramatic decrease of reset resistance was observed after *ca.* 160 cycles which indicated the failure of this device. This is a typical “stuck-set” failure in phase-change memory, which is normally caused by either the heater seasoning or stoichiometric shifts of the phase-change material [28]. The Ge_2.4_Sb_2.0_Te_5.6_ memory cell shows a similar performance. Substantial improvements of endurance performance can be observed for the Ge_3.5_Sb_1.0_Te_5.5_- and Ge_5.0_Sb_3.5_Te_1.5_-based memory cells, where the number of cycles increases to over 400 and 1000, respectively. However, it should be noted that this improvement of endurance comes with a decrease in the resistance ratio as a trade-off.

**Fig. 7:**
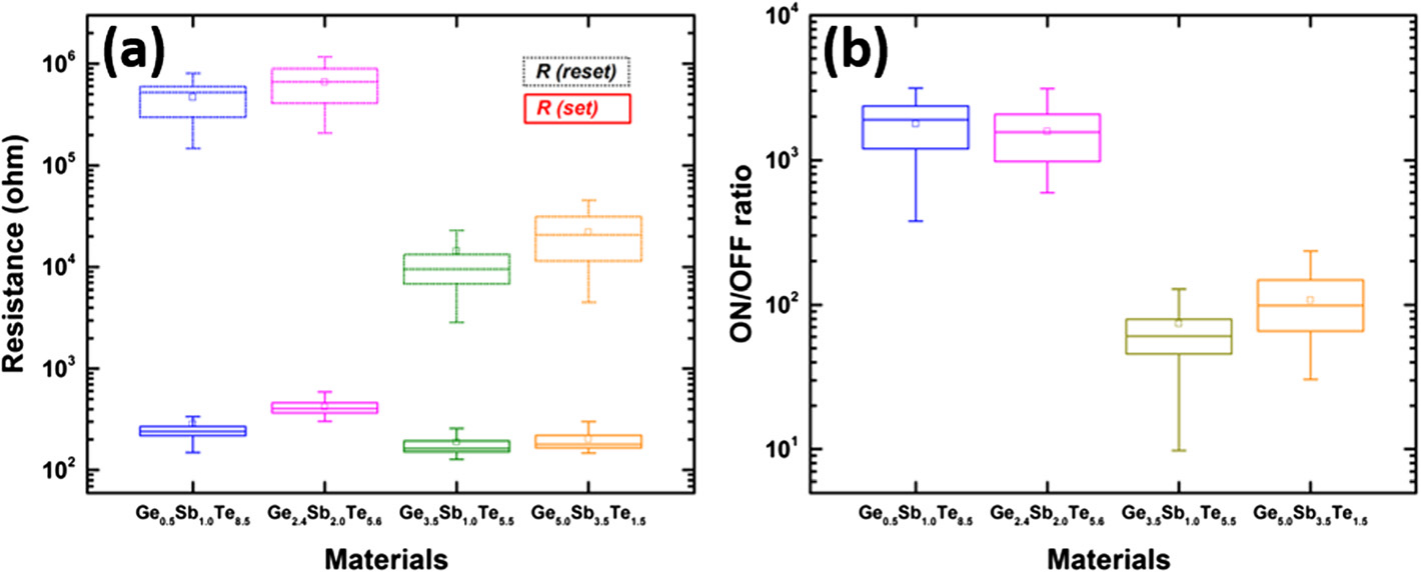
Distributions of (**a**) the resistances at both set and reset states and (**b**) resistance ratio for the four memory cells. *Box plot* shows the median, first and third quartiles. The *whiskers* show the data points that are beyond the quartiles by one and a half interquartile range. The *small box* shows the average value

The distribution of the resistance for the four memory cells at both set and reset states is illustrated in the box plot in Fig. 7a. All four resistances in the set state are similar in value and show relatively small variation. However, resistances in the reset state are characterised by larger differences and variation, especially for the Ge_5.0_Sb_3.5_Te_1.5_-based memory cell. This leads to the differences in the resistance ratio as shown in Fig. 7b. A large resistance ratio of reset and set states (*ca.* 10^3^) is observed for both Ge_0.5_Sb_1.0_Te_8.5_- and Ge_2.4_Sb_2.0_Te_5.6_-based memory cells. For Ge_3.5_Sb_1.0_Te_5.5_- and Ge_5.0_Sb_3.5_Te_1.5_-based memory cells, the resistance ratios drop to *ca.* 10^2^ due to the lower reset resistances.

## Conclusions

The phase-change memory properties of electrodeposited GeSbTe thin films have been measured for various compositions. The composition of the resultant films can be tuned by varying the deposition potential in a single electrolyte, making their future application in depositing super-lattice structures possible. Film composition modulation was also achieved by varying the electrolyte concentrations with deposition of four device-quality GeSbTe thin films (Ge_0.5_Sb_1.0_Te_8.5_, Ge_2.4_Sb_2.0_Te_5.6_, Ge_3.5_Sb_1.0_Te_5.5_ and Ge_5.0_Sb_3.5_Te_1.5_). Phase-change memory cells based on the four films have shown promising switching properties with high-resistance ratio (three orders of magnitude) and good durability.
